# Effect of deep margin elevation with different base materials on periodontal health in a clinical study

**DOI:** 10.1038/s41598-026-61409-6

**Published:** 2026-07-16

**Authors:** Rofida Ragab, Ahmed Abdallah, Rasha Saad, Mona Riad

**Affiliations:** 1 Conservative Dentistry Department, Faculty of Dentistry, Minia University, Minia, Egypt; 2 Oral Diagnosis and Periodontology Department, Faculty of Dentistry, Minia University, Minia, Egypt; 3 Conservative Dentistry, Faculty of Dentistry, Cairo University, Cairo, Egypt

**Keywords:** Gingival margin elevation, Bleeding on probing, Probing depth, Periodontal health, Diseases, Health care, Materials science, Medical research

## Abstract

This prospective clinical investigation evaluated the effects of proximal box elevation using three base materials on periodontal tissues in premolar teeth treated with direct composite restorations. In endodontically treated maxillary premolars, gingival crevicular fluid (GCF) samples were collected after restoring cavities using three base materials: resin-modified glass ionomer (RMGI), flowable composite (Tetric N-Flow), and injectable hybrid composite (Beautifil Flow Plus). Periodontal health was assessed by measuring interleukin-1 (IL-1) and tumor necrosis factor (TNF) concentrations, bleeding on probing (BOP), and pocket depth (PD) immediately and at 3 months postoperatively. Data were statistically analyzed using one-way ANOVA to compare groups, Levene’s test to assess homogeneity of variances, and paired t-tests and chi-square tests were used to assess differences within groups. the results revealed that the levels of IL-1, TNF, and PD differed significantly among the three base materials at different time points. The Beautifil Flow Plus group showed the highest TNF levels, the most substantial absolute reduction, and the lowest reduction in IL-1 levels. However, there were no significant differences in BOP among the three materials at any time interval. Within the limitations of this three-month study, all materials were clinically acceptable, though Tetric N-Flow and RM-GI showed a more favorable short-term inflammatory profile than Beautifil Flow Plus. However, due to the limited sample size and short follow-up, no definitive claims regarding long-term periodontal compatibility or clinical superiority can be made.

**Supplementary Information:**

The online version contains supplementary material available at 10.1038/s41598-026-61409-6.

## Introduction

Dental restorations maintain periodontal health, which is crucial for the long-term success of interproximal restorations^1^. For dentists, treating large carious lesions that jeopardize subgingival margins and interproximal contacts is a major challenge, as these lesions interfere with multiple stages of the process required to achieve the best possible restorations. Effective caries excision, cavity preparation, impression making, rubber dam isolation, gingival bleeding control, appropriate restorative application, removal of excess cement, and biological width compliance are all hampered by deep cervical lesions. The lifespan of the restoration is reduced when saliva, blood, or gingival crevicular fluid contaminates the tooth surface and the restoration margin contact^2^.

The main determinant of periodontal health is biological width. Significant cervical defects can lead to periodontal deterioration, inflammation, gingival recession, osseous loss, and bleeding, and their treatment frequently depends on the biological width^3^. The biological width is the measurement of the soft tissue associated with the tooth segment located above the alveolar bone crest. One of the most important surgical procedures for maintaining biological width is crown lengthening. Gingivectomy is a supplemental procedure believed to improve procedural efficiency, reposition the margin, and create a favorable environment for the periodontium. Longer treatment times, higher costs, patient discomfort, reduced dental aesthetics, attachment loss, and proximity to root concavities and furcation areas are among the disadvantages of surgical crown lengthening^4,5^.

Deep margin elevation (DME) is a procedure that elevates or repositions subgingival margins to supragingival positions utilizing various materials to enhance marginal integrity and bonding strength. In 1998, Dietschi and Spreafico presented the DME procedure to mitigate complications related to subgingival restorations. Nevertheless, it remains a relatively innovative methodology. Currently, clinical dentistry promotes conservative and, in some instances, minimally invasive dental management techniques as alternatives to the invasive procedure of crown lengthening, which can lead to less trauma and faster recovery for patients^6^.

The placement of subgingival margins is associated with detrimental inflammatory periodontal responses resulting from insufficient tooth-restoration interfaces, excessive restoration contouring, challenges in maintaining oral hygiene, heightened pathogenicity of subgingival dental plaque, and a breach of the biologic width principle. Clinical and histological investigations indicate that subgingival restorative margins may have detrimental effects on tissues, despite adequate control of bacterial plaque. Localized gingival inflammation, elevated plaque and gingival index scores, and increased probing depths have been observed around prostheses with subgingival margins, in contrast to those with supragingival margins in the natural dentition^7^.

Proinflammatory cytokines play a role in vascular dilation, increased permeability, and inflammatory response. Since periodontal diseases are inflammatory, proinflammatory cytokines have been implicated in local tissue destruction, and their levels have been reported to be increased in both saliva and GCF^8^.

The concentration of GCF cytokines accurately reflects events in dental tissues. Their importance must be assessed in relation to tooth alignment and the severity of carious lesions, particularly the differential effects of dental restoration materials on GCF IL-1 and TNF, which are indicators of localized inflammation^9^. IL-1 is a pivotal factor in the etiology of carious lesions.

The direct composite restoration of teeth is a straightforward, economical, and dependable solution for various clinical scenarios. Progress in adhesive technology, the introduction of modern materials, heightened aesthetic expectations, and the need for minimally invasive techniques have led to the extensive application of adhesives in modern dentistry. The application of adhesive materials in the subgingival region is complicated, as overhanging margins are crucial for preserving periodontal tissues. The placement of the cervical margin of restorations is critical, as it may adversely affect biofilm accumulation, irritate gingival tissues, and potentially infringe upon the biological width^10^.

The open-sandwich method, which aims to address sealing issues in deep Class II direct composite restorations, is considered a forerunner of DME. It fills the cervical area of the cavity with glass ionomer or resin-modified glass ionomer (RMGI), leaving some of the material exposed to the oral environment^11^.

The success of this technique depends heavily on the biomechanical stability of the elevation material. Highly filled, flowable composites have become an accepted standard for DME; their low viscosity enables excellent adaptation to the gingival floor, while their physical properties provide a stable base that withstands polymerization stresses. Flowable composites (e.g., Tetric N-Flow) may also serve as stress absorbers or stress breakers. Stress absorption is strongly influenced by the elastic modulus and other physical properties of restorative materials^12^.

Laboratory studies have validated the use of resin-modified glass ionomer (RMGI) for this purpose, citing its hydrophilic nature, fluoride release, and chemical bond to moist dentin as critical to achieving a predictable cervical seal^13^.

Furthermore, advances in materials science have introduced injectable hybrid composites, such as Beautifil Flow plus, that combine the adaptability of a flowable composite with the mechanical strength of a traditional hybrid. Research indicates that these materials provide a tight marginal seal and high radiopacity, both of which are essential for long-term radiographic monitoring, and have the capacity to release fluoride^14^.

The effects of proximal box elevation on the periodontal tissues of premolar teeth filled with direct resin composite were investigated in this study using three base materials: an injectable hybrid composite, a flowable composite, and a resin-modified glass ionomer. The null hypothesis is that there is no discernible difference in deep margin elevation among the periodontal tissues of direct resin composite restorations when using injectable hybrid composite, RMGI, or flowable composite base materials.

## Materials and methods

### Study design

A randomized clinical study assigned participants to three groups (e.g., a recently introduced injectable resin composite, a standard treatment with RM-GI, and a flowable composite) to fairly compare effectiveness. All participants underwent thorough periodontal and radiographic evaluations immediately postoperatively and at 3-month follow-up.

### Protocol registration and ethical approval

The protocol for this study was registered on ClinicalTrials.gov (http://www.clinicaltrials.gov) with ID NCT06944080 (16/04/2025). This randomized controlled clinical trial followed the Consolidated Standards of Reporting Trials (CONSORT) guidelines. This experimental protocol and all investigations involving human subjects were approved by the Research Ethics Committee, Faculty of Dentistry, Minia University, under code number 475 (Ref. no. 02/12/2024), in accordance with the Declaration of Helsinki. All experiments were conducted in accordance with relevant guidelines and regulations.

### Sample size calculation

Three sets of twelve samples each made up the study’s total sample size of *N* = 36. Power exceeded 80%, and the significance threshold was set at 0.05. With a value of 0.5166, the sample size was sufficient, and the confidence interval was 95%. G*Power version 3.1.9 was used to determine the sample size^15^.

### Patient selection

Clinical trial participants were recruited from the Conservative Clinic at Minia University’s Faculty of Dentistry, which regularly serves a large patient population that meets the inclusion criteria. Eligible patients were identified according to the participant schedule. The lead investigator obtained informed consent and explained all aspects of the study to eligible volunteers. All participants provided written informed consent before enrollment, after being fully informed about the study’s background, including its goals, specific protocols (such as the number of visits), possible advantages, and potential drawbacks. Males and females 18 years of age or older who demonstrated adequate oral hygiene, periodontal probing depths of ≤ 4 mm, full-mouth plaque scores (FMPS), bleeding scores (FMBS) of ≤ 20%, and at least 2 mm of keratinized tissue were eligible to participate^16^. Participants with disabilities, systemic diseases, or serious medical conditions, such as heart disease, diabetes, hypertension, epilepsy, or hematological disorders, as well as those allergic to any research ingredient, were excluded. Patients with severe or active periodontal disease, those who had received therapeutic radiation to the craniofacial region, those who were unable to attend follow-up appointments, and those who had participated in a clinical trial within 3 months of the trial’s start were all excluded^16^.

### Randomization, allocation of participants, and concealment

A parallel-group randomized clinical trial of patients with deep subgingival interproximal carious lesions employed simple randomization (1:1:1 ratio) to assign participants, using computer-generated numbers (https://www.random.org/) to reduce selection bias, as demonstrated by Sil et al. in 2019^17^. The random sequence was generated by an independent researcher not involved in the clinical treatment. Participants were assigned in a 1:1:1 ratio to one of three groups: Group 1: Resin-Modified Glass Ionomer (RMGI). Group 2: Tetric N-Flow (Flowable Composite). Group 3: Beautifil Flow Plus (Injectable Hybrid Flowable). To ensure allocation concealment, sequentially numbered, opaque, sealed envelopes were used. After the enrolling investigator confirmed eligibility and obtained informed consent, the participant selected an envelope to determine their group. The assigned number was then recorded in the patient’s chart.

### Blinding

Due to the distinct physical properties and application protocols of the restorative materials used for deep marginal elevation (resin-modified glass ionomer, flowable composite, and injectable hybrid flowable composite), neither the patients nor the operator were blinded. To reduce performance and detection bias, laboratory personnel performing the biochemical analysis were blinded to the treatment groups to eliminate detection of cytokine levels; two knowledgeable assessors were also blinded to each patient’s treatment assignment. Additionally, the statistician assigned to analyze the findings was blinded.

Preoperative evaluation protocols were selected for patients with deep subgingival interproximal carious lesions. Dental plaque was removed before evaluation, as there is a link between carious lesions and plaque buildup. A fluoride preventive paste and a bristle brush were used for dental prophylaxis, including scaling and polishing. Using a calibrated periodontal probe, a preoperative photo, and pocket depth measurements were obtained (Nordent Manufacturing Inc., USA).

### Clinical procedures

Patients received 1.4 ml of 2% lidocaine with 1:2,500 phenylephrine (SS White 100) via field block, plus 0.3 ml into the buccal/palatal papilla. Class II mesio-occlusal or disto-occlusal inlay cavities were prepared using high-speed diamond (881.31.014 FG; Brasseler) and carbide (SF-41; Mani) burs under water cooling. Carious tissue was removed until the gingival floor reached 2 mm below the cementoenamel junction^18^.

**Fig. 1:**
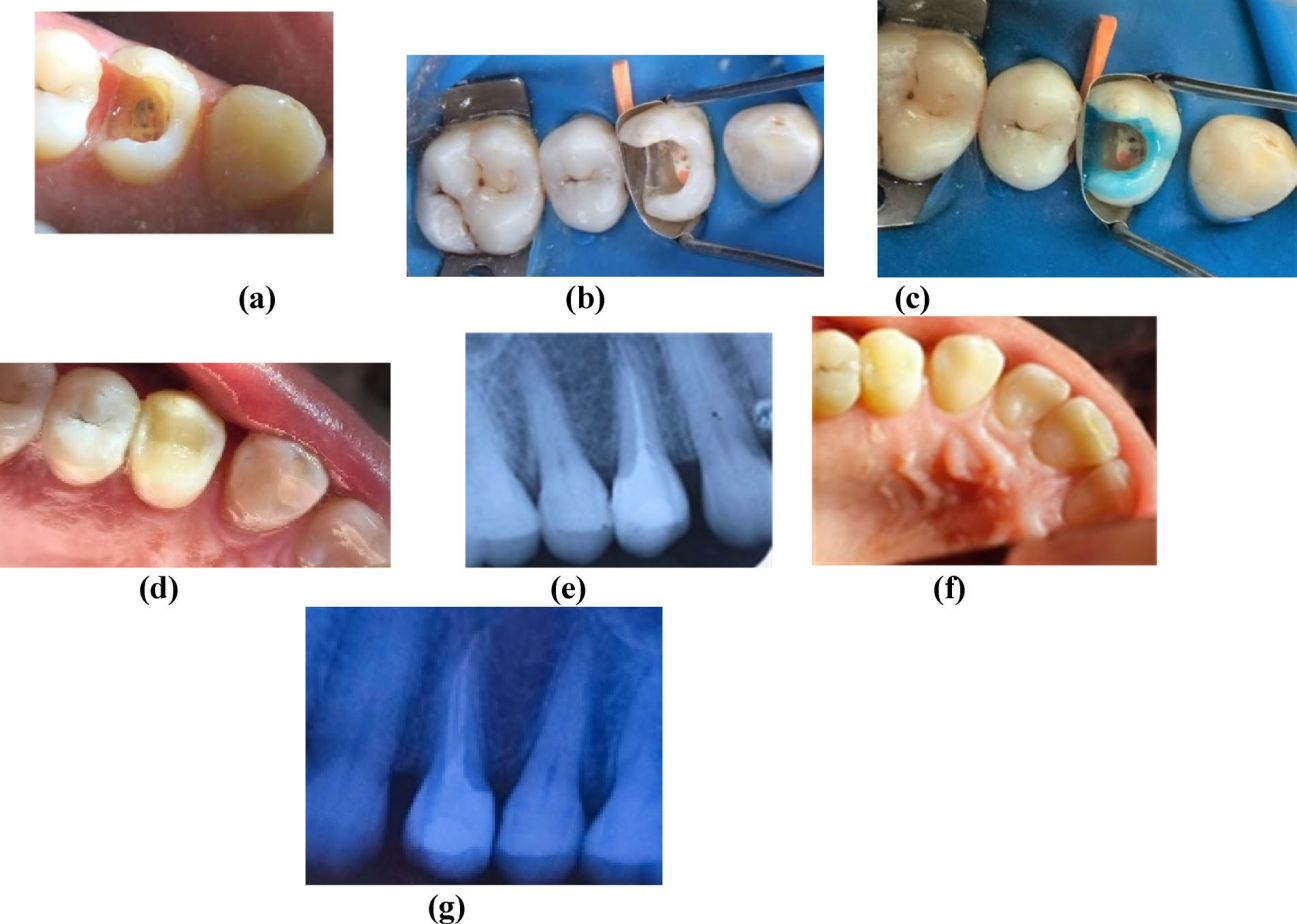
Steps of preparation, restoration, and 3 months of follow-up. (**a**) Cavity preparation, (**b**) rubber dam isolation, matrix, and wedge placement, (**c**) restorative procedure, (**d**) after final restoration, (**e**) radiograph after final restoration, (**f**) clinical photo after 3 months of follow-up g-radiograph after 3 months of follow-up.

Deep margins were elevated using the base materials. Teeth were isolated with a rubber dam (OptraDam Plus, Ivoclar Vivadent) and contoured using a saddle-shaped matrix (TOR-VM) and flexible wedges (Garrison/Bioclear)^19^, as depicted in Fig. 1.

The materials employed in this study are specified in Table 1.

### Base materials application

*RMGI Base*: Dentin was conditioned (GC Fuji Plus Conditioner) for 20s, rinsed, and dried. RMGI (GC Fuji Plus) was mixed for 10s (Ultramat2), applied to the gingival floor, and light-cured for 20s at 1200 mW/cm².

*Composite Base*: For the flowable (Tetric N-Flow) or injectable hybrid groups (Beautifil Flow Plus), enamel margins were etched with 37% phosphoric acid for 30s, rinsed, and dried. Adhesive was agitated for 15 s, air-thinned, and light-cured for 20 s before the base was injected and light-cured for 20 s^20^.

### Restorative procedures

Following base placement, enamel margins were etched with 37% phosphoric acid for 15s, rinsed, and dried. Adhesive was applied until a stable film was achieved and light-cured for 20s. A nanoceramic composite was placed incrementally, with a focus on the proximal surfaces. Each layer was light-cured for 40s. Anatomical contours were refined using fine-grit diamond burs and flexible discs (Sof-Lex, 3 M ESPE) under water cooling. Final polishing was performed with discs, brushes, and diamond paste (Prisma Gloss, Dentsply)^21^.

The clinical performance of the restorations (marginal adaptation, surface integrity) was evaluated according to the modified USPHS criteria. USPHS criteria for marginal adaptation were as follows: Alpha: Explorer does not catch; no visible crevice. Bravo: Explorer catches, but no visible crevice or dentin exposure. Charlie: The explorer penetrates a crevice to a depth that exposes dentin. Delta: Immediate replacement is necessary due to structural failure or secondary caries. While the USPHS criteria for surface integrity were as follows: Alpha: The surface is as smooth as the surrounding enamel; Bravo: Slightly rough or pitted but acceptable; Charlie: The surface is deeply pitted with irregular grooves; Delta: Beyond repair, requires immediate replacement.

### Assessment of gingival and periodontal health

Periodontal health was assessed by measuring the pocket depth at the proximal regions of the restorations using a periodontal probe (PCPUNC 15, Hu-Friedy; Chicago, IL, USA). Bleeding on probing (BOP) at designated sites was recorded as binary (“BOP: yes”; “BOP: no”). Bleeding on probing (BOP) was utilized to evaluate periodontal tissue inflammation. The presence or absence of bleeding was recorded as a binary outcome (“yes” or “no”) at the designated sites of the test and control teeth. The collected BOP data were subsequently expressed as percentages and subjected to inferential statistical analysis to evaluate the clinical periodontal response^22^.

### Clinical performance assessment

All steps of the restoration were achieved via a single experienced operator. The clinical performance of the restorations was clinically assessed by two other calibrated, experienced, blinded examiners. Follow-up was performed during the immediate postoperative period and at 3 months, using magnifying eye loupes. Further, the assessment was conducted using photographs at each follow-up visit, from the immediate postoperative period to the end of the follow-up period. In the event of any disagreements during the evaluation, the two evaluators reached a consensus to make a single final decision.

### Collection of samples for the measurement of biological markers

Samples for biological marker measurement were obtained from the proximal surfaces of each tooth adjacent to the restored region. Gingival crevicular fluid (GCF) samples were collected utilizing standard-sized paper strips (Periopaper, OraFlow Inc., NY, USA) following the isolation of tooth peripheries with rolled cotton pads to avert saliva contamination. The paper strips were inserted into the sulcus to a maximum depth of 1 mm and maintained for 30 s until the patient felt mild pressure. Strips tainted with blood were omitted from the study. The paper strips were then placed in Eppendorf tubes containing 1000 µl of saline using an automatic pipette (Pipette, Borox, BeyanLab, Istanbul, Turkey) and stored at -20 °C until biochemical analysis .

### Biochemical evaluations (ELISA assays)

Commercial kits were used to measure the proinflammatory mediators TNF and IL-1 in GCF samples stored at -20 °C. Before the workday, samples stored at -20 °C were thawed for 24 h at + 4 °C. Before analysis, samples were centrifuged at 3000 rpm for 10 min (SK962, SinoThinker, Shenzhen, China) and vortexed for 1 min (Vortex, Velp Scientifica, Italy) to extract GCF from the Periopapers. Following the manufacturer’s instructions, ELISA kits (Sunred Biotech Co., Shanghai, China) were used to measure TNF and IL-1 levels. Each mediator’s specific antibody was used as a pretreatment on microdilution plates. The specified testing procedures were carried out using commercial kits that employ the Streptavidin-HRP double-antibody sandwich approach, in accordance with the manufacturer’s instructions^22^ (Tables 2, 3).

**Table 1:** Materials, specifications, chemical composition, manufacturers, and Batch numbers.

Material used	Specification	Composition	Manufacturer	Batch number
Tetric N-Flow	Flowable-composite	36 wt% dimethacrylate (including TEGDMA), 63 wt.%fillers (barium glass, ytterbium trifluoride, highly dispersed silica, and mixed oxide), and 1wt.%initiator, stabilizers, and pigment	Ivoclar Vivadent, Schaan, Liechtenstein, USA https://www.ivoclar.com	Z04360
N-Etch	Etchant agent	Etchant agent.		Z014VZ
Tetric N- Bond Universal	One-step self-etch adhesive system	Water, ethanol, methacrylates, highly dispersed silicon dioxide, stabilizers, and initiators.		Z00SNR
GC Fuji II LC capsule	Resin-modified glass ionomer	Liquid: methacrylate, water, polyacrylic acid, dimethacrylate, carboxylic acid, initiator, stabilizer -powder: fluoro-alumino-silicate glass, initiator, pigment	76 − 1 Hasunuma-cho, Itabashi-ku, Tokyo 174–8585, Japan (GC Co., Tokyo, Japan)	240,307 A
Beautifil Flow PLUS	Flowable-composite	Bis-GMA^1^, TEGDMA^2^, S-PRG ^3^filler based on fluoroboroaluminosilicate, polymerization initiator, pigments, and others	SHOFU INC 11kamitakamatsu-cho, Fukuine, Higashiyama-ku, Kyoto 605–0983, Japan http://www.shofu.com	122,219
SPECTRA ST	Universal composite restorative	Methacrylate-modified polysiloxane (organically modified ceramic) dimethacrylate resins, ethyl-4-(dimethylamino)benzoate, and bis(4-methylphenyl)iodonium hexafluorophosphate. Filler load: 78–80% by weight: Spherical, pre-polymerized SphereTEC fillers (d3,50 ≈ 15 μm), non-agglomerated barium glass, and ytterbium fluoride	Dentsply DeTrey GmbH De-Trey-str. 178,467 Konstanz Germany http://www.dentsplysirona.com	221,100,178

1-Bisphenol-A glycidyl ether dimethacrylate 2-Tri-ethylene glycol dimethacrylate, 3-Surface Pre-Reacted Glass-ionomer.

### Statistical analysis

Statistical analyses were performed using IBM SPSS Statistics software (version 26; IBM Corp., Armonk, NY, USA).

1. Data Description and Assumption Testing:
  *Descriptive Statistics*: Continuous variables were summarized as mean, standard deviation (SD), and range. Categorical (nominal) variables were expressed as frequencies and percentages.
  *Normality Testing*: The distribution of continuous data was assessed using normality tests to confirm the validity of parametric assumptions.
  *Homogeneity of Variances*: Levene’s test was used to assess the equality of variances across groups. The assumption of homogeneity was satisfied when *p* > 0.05.
2. Inferential Statistics and Group Comparisons. To ensure clarity, data comparisons were divided based on the variable type and study timeline:
  Categorical Variables:
  Between-Group Comparisons: The Chi-square test was used to evaluate differences in categorical distribution among the study groups.
  Continuous Variables (Parametric Data):
  Between-Group Comparisons: A one-way Analysis of Variance (ANOVA) was applied to compare the mean values across the multiple study groups.
  Post-Hoc Pairwise Comparisons: Following a significant ANOVA result, Tukey’s multiple-comparison test was performed to identify significant differences between specific pairs of groups.
  Within-Group Longitudinal Comparisons: A paired t-test was utilized within each group to analyze the changes over time, specifically comparing immediate post-o measurements to the 3-month follow-up.
3. Significance Level.
  For all statistical tests, a p-value < 0.05 (*) was considered statistically significant, and a p-value < 0.001 (**) was considered highly significant.

## Result

**Table 2:** Demographic data for the studied group.

Variable	Tetric *N*-Flow group	RM-GI	Beautifil Flow Plus group	*p*-value
Age (years)				
Mean ± S.D	29.17 ± 5.73	29 ± 6.03	28.83 ± 5.64	0.990
Min–max	19–38	22–39	18–37	
Gender				
Male	6(50%)	6(50%)	5(41.7%)	0.895
Female	6(50%)	6(50%)	7(58.3%)	

There is a significant difference at P-value < 0.05 (*), and a highly significant difference at P-value < 0.001 (**).

### Normality test

**Table 3:** Shapiro-Wilk normality results for study variables across groups.

Groups	TNF (pg/ml)	IL1B (pg/ml)	Pocket depth (mm)
	Shapiro-Wilk (*p*-value)	Shapiro-Wilk (*p*-value)	Shapiro-Wilk (*p*-value)
Tetric N-Flow group	0.960 (0.809)	0.957 (0.789)	0.883 (0.325)
RM-GI group	0.900(0.411)	0.878(0.302)	0.821 (0.119)
Beautifil Flow Plus group	0.953(0.757)	0.937(0.646)	0.883(0.325)

There was a significant difference at P-value < 0.05 (*), and a highly significant difference at P-value < 0.001 (**).

This table indicates that the data were normally distributed (Table 4).

**Table 4:** Comparison of TNF results across the study groups at different time points.

Tumor necrosis factor (TNF)								
Groups		Immediately postoperative			Follow-up in 3 months			Paired t-test
		Mean ± S.D	95% CI	Min—Max	Mean ± S.D	95%CI	Min—Max	T (P-value)
Tetric N-Flow group		23.02 ± 2.23	21.60-24.44	20.51–26.32	19.00 ± 2.07	17.68–20.32	17.08–21.93	14.701 *p*<0.0001**
RM-GI group		35.79 ± 2.51	34.20-37.38	32.06–38.04	23.54 ± 1.47	22.61–24.47	21.62–25.12	22.299 *p*<0.0001**
Beautifil Flow Plus group		76.05 ± 5.84	72.34–79.67	67.25–82.14	38.57 ± 3.09	36.61–40.53	35.06–42.56	23.166 *p*<0.0001**
One-way ANOVA	F(P-value)	253.102 (*p* < 0.0001**)			98.552(*p* < 0.0001**)			--------

There is a significant result at P-value < 0.05 (*), and highly significant result at P-value < 0.001 (**).

Levene’s test confirmed homogeneity of variances for TNF levels at both time points (immediately postoperative: *p* = 0.109; at 3 months: *p* = 0.237), supporting the use of one-way ANOVA.

**Fig. 2:**
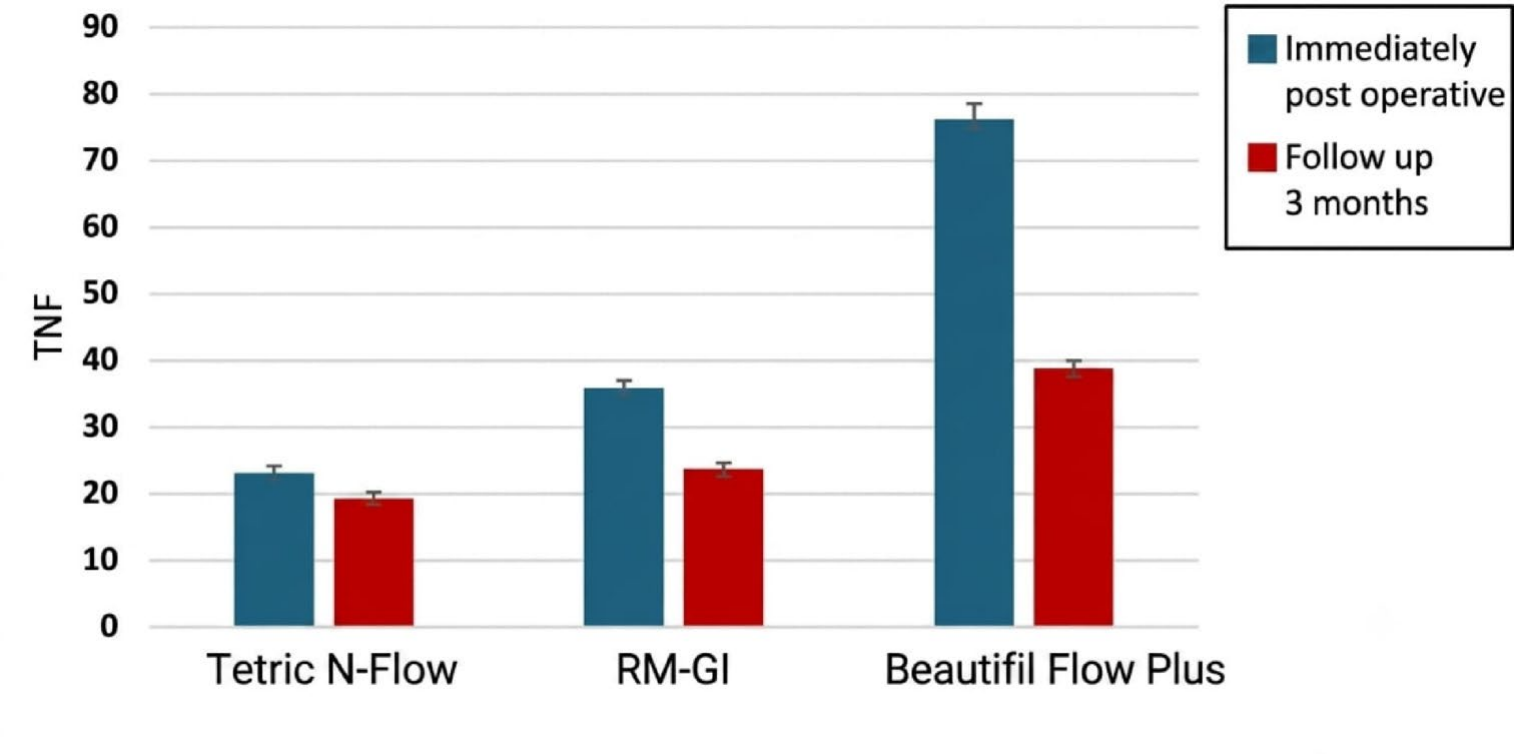
Comparison of TNF results across the study groups at different time points.

All materials showed a statistically significant decrease in TNF levels (*p* < 0.001) from the immediate postoperative period to the three-month follow-up (Fig. 2, Table 5).

**Table 5:** Multiple post hoc pairwise comparisons of TNF (pg/ml) levels across study groups at different time points.

Durations	Tetric *N* Flow vs. RM-GI	Tetric *N*-Flow vs. Beautifil Flow plus	RM-GI vs. Beautifil Flow Plus
Immediately postoperative	*p* < 0.001	*p* < 0.001	*p* < 0.001
Follow-up in 3 months	*p* < 0.023	*p* < 0.001	*p* < 0.001

There is a significant difference at *p* < 0.05 (*), and a highly significant difference at *p* < 0.001 (**).

Tukey’s post-hoc analysis indicated that the type of material used had a significant impact on TNF levels at both evaluation periods. Beautifil Flow Plus consistently exhibited a highly significant difference (*p* < 0.001) in TNF levels, with noticeably higher levels compared to both Tetric N-Flow and RMGI across all intervals (Tables 6, 7).

**Table 6:** Comparison of IL1B pg/ml results across the studied groups at different time points.

Interleukin 1 (IL1) pg/ml								
Groups		Immediately postoperative			Follow-up in 3 months.			Paired t-test
		Mean ± S.D	95% CI	Min-Max	Mean ± S.D	95% CI	Min-Max	T (P-value)
Tetric N-Flow group		27.49 ± 2.13	26.14–28.84	25.13–30.34	18.59 ± 1.63	17.55–19.63	16.78–20.56	36.838 *p*<0.0001**
RM-GI group		36.92 ± 2.01	35.64–38.20	34.45–39.08	22.66 ± 0.437	22.38–22.94	22.16–23.21	18.544 *p*<0.0001**
Beautifil Flow Plus group		46.81 ± 4.25	44.14–49.51	41.92–51.86	30.31 ± 1.88	29.11–31.51	27.63–32.49	11.453 *p*<0.0001**
One-way ANOVA	F(P-value)	52.603 (*p* < 0.0001**)			83.411 (*p* < 0.0001**)			--------

There is a highly significant result at P-value < 0.001 (**).

Levene’s test confirmed homogeneity of variances for IL1B pg/ml levels at both time points (immediately postoperative: *p* = 0.078; at 3 months: *p* = 0.057), supporting the use of one-way ANOVA.

**Fig. 3:**
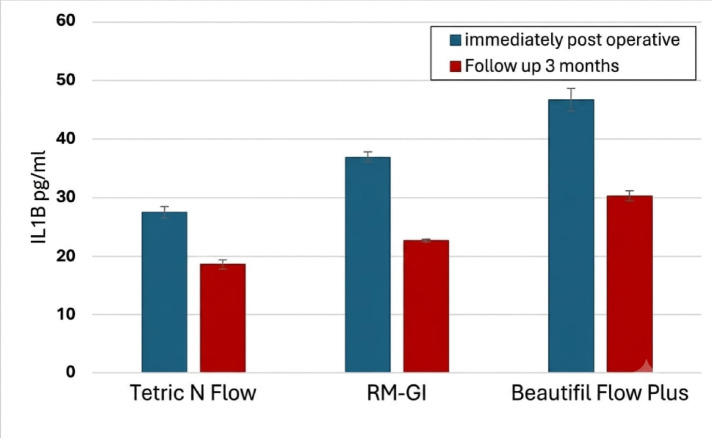
Comparison of IL-1β pg/ml results across the studied groups at different time points.

Comparative Analysis (Intra-group): All studied materials demonstrated a statistically significant, consistent reduction in inflammation over the three months (*p* < 0.0001). Tetric N-Flow exhibited the largest absolute decrease in IL-1β levels, while Beautifil Flow Plus maintained the highest inflammatory values immediately postoperative and at follow-up (Fig. 3).

**Table 7:** Multiple post hoc pairwise comparisons of IL-1β (pg/ml) levels across study groups at different time points.

Durations	Tetric *N*- Flow vs. RM-GI	Tetric *N* -Flow vs. Beautifil Flow Plus	RM-GI vs. Beautifil Flow Plus
Immediately postoperative	*p* < 0.001	*p* < 0.0001	*p* < 0.0001
Follow-up in 3 months	*p* < 0.002*	*p* < 0.0001	*p* < 0.0001

There is a significant difference at *p* < 0.05 (*), and a highly significant difference at *p* < 0.001 (**).

Inter-group post hoc comparisons revealed highly significant differences across all material pairs at both time points. The comparison of Beautifil Flow Plus against both Tetric N-Flow and RMGI demonstrated a highly significant difference (*p* < 0.0001) immediately postoperatively and at 3 months, indicating that Beautifil Flow Plus elicits a markedly higher inflammatory response than the other two materials (Table 8).

**Table 8:** Comparison of bleeding-on-probing results across the groups studied at different time points.

Bleeding on probing						
Durations		Immediately postoperative		Follow- up in 3 months		Chi-square
		Yes	No	Yes	No	$\:chi-square$ (P-value)
Tetric N-Flow group		(100%)	(0%)	(60%)	(40%)	2.500 (0.114)
RM-GI group		(100%)	(0%)	(60%)	(40%)	2.500 (0.114)
Beautifil Flow Plus group		(100%)	(0%)	(60%)	(40%)	2.500 (0.114)
Chi-square	$\:chi-square$ (P-value)	0.0001 (1.000)		0.0001 (1.000)		–

Immediately postoperatively, all clinical cases across all groups exhibited 100% bleeding on probing, showing no statistically significant difference (*p* = 1.000).

**Fig. 4:**
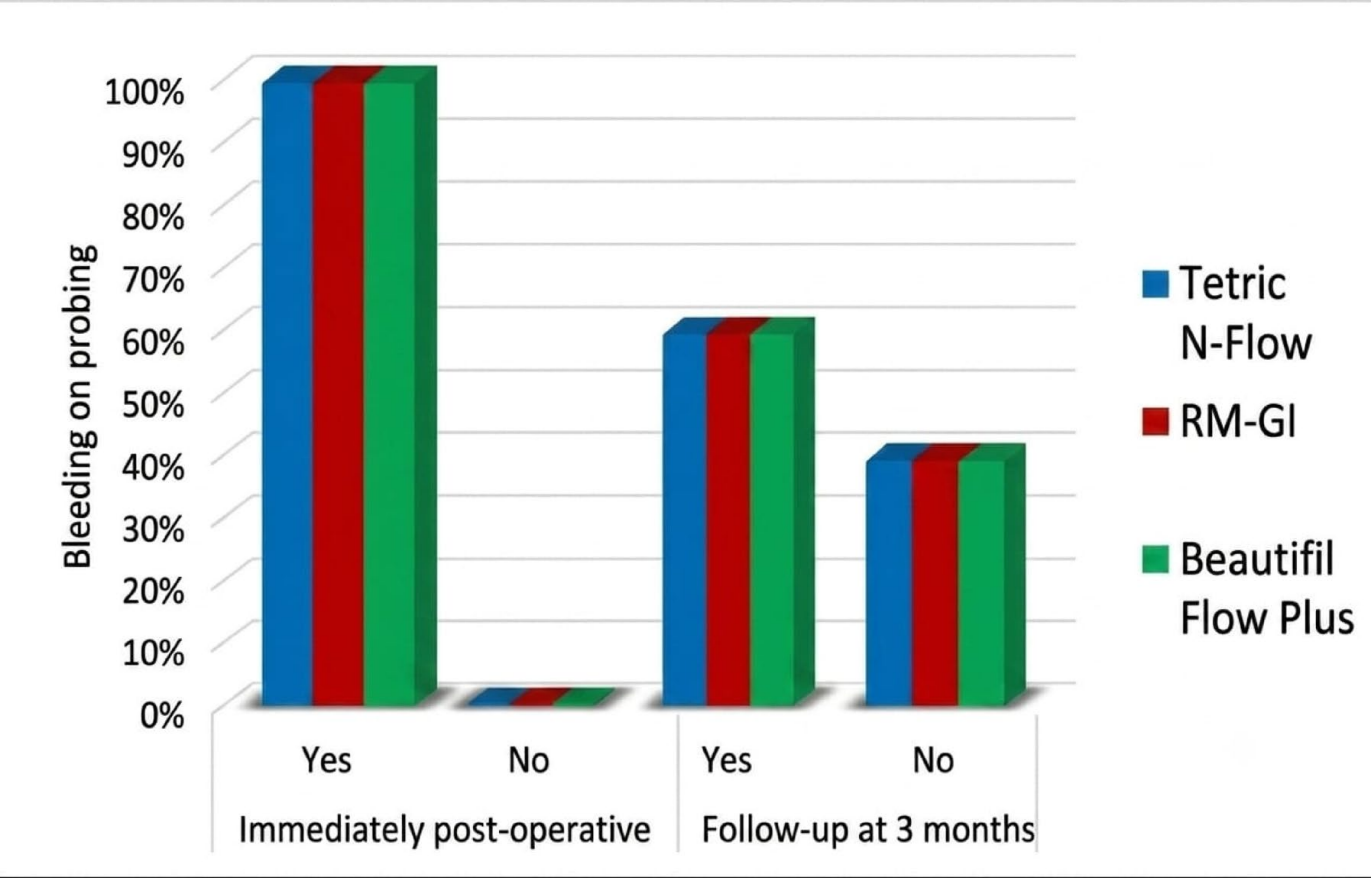
Comparison of bleeding-on-probing results across the groups studied at different time points.

At the 3-month follow-up, the percentage of cases presenting with positive BOP decreased to 60% in the Tetric N-Flow, RM-GI, and Beautifil Flow Plus groups, while 40% of the cases achieved cessation of bleeding. The Chi-square test revealed no statistically significant differences in BOP rates among the three restorative materials at this follow-up interval (*p* = 1.000) (Fig. 4, Table 9).

**Table 9:** Comparison of pocket depth results across the studied groups at different time points.

Pocket depth								
Groups		Immediately postoperative			Follow-up in 3 months			Paired t-test
		Mean ± SD	95% CI	Min-Max	Mean ± SD	95% CI	Min-Max	T (P-value)
Tetric N-Flow group		3.40 ± 0.548	3.05–3.75	3–4	2 ± 0.71	1.55–2.45	1–3	5.715 *p* < 0.005*
RM-GI group		3 ± 1	2.36–3.64	2–4	1.60 ± 0.55	1.24–1.95	1–2	5.715 *p* < 0.005*
Beautifil Flow Plus group		3 ± 0.707	2.45–3.55	2–4	1.60 ± 0.55	1.25–1.95	1–2	*p* < 0.005*
One-way ANOVA	F(P-value)	0.444 (0.651)			0.727(0.503)			--------

There is a significant at P-value < 0.05 (*), and highly significant at P-value < 0.001 (**).

Levene’s test confirmed homogeneity of variances for pocket depth at both time points (immediately postoperative: *p* = 0.305; at 3 months: *p* = 0.907), supporting the use of one-way ANOVA.

### Intragroup comparison (immediately postoperative versus three-month follow-up)

**Fig. 5:**
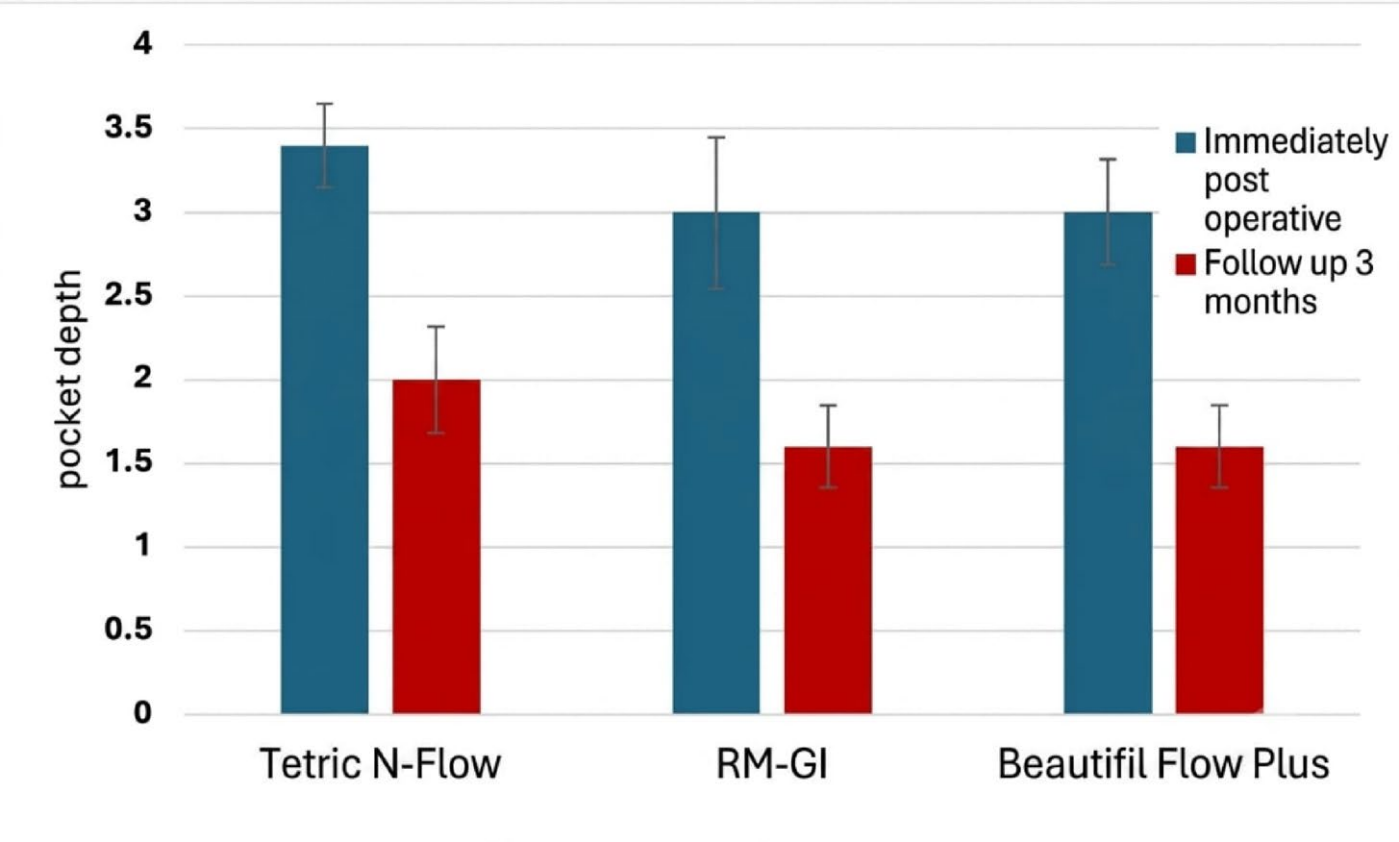
Comparison of pocket depth results across the studied groups at different timepoints.

While all materials significantly reduced pocket depth after three months, no single substance exhibited a distinctly superior efficacy relative to the others (Fig. 5).

### Clinical evaluation result

Clinical evaluation using the USPHS criteria revealed that 100% of the restorations remained clinically acceptable at the 3-month follow-up, with all parameters scoring either ‘Alpha’ or ‘Beta’. Because no clinical failures or significant degradation occurred during this short-term period, statistical analysis was not performed on these descriptive, immediately postoperative safety data.

## Discussion

The present study evaluated the impact of three restorative materials (RMGI, Tetric N Flow, and Beautifil Flow Plus) on periodontal health during Deep Margin Elevation (DME). Our findings rejected the null hypothesis, as significant differences in cytokine levels (TNF-α and IL-1) and clinical parameters were observed between the groups, with Beautifil Flow Plus consistently exhibiting the highest inflammatory marker levels.

In the current study, all materials triggered an immediate postoperative increase in GCF proinflammatory cytokines (IL-1 and TNF), followed by a significant reduction at the 3-month follow-up. This aligns with Stefanovic et al.^23^, who suggest that placement of restorative materials and the subsequent healing process may temporarily activate local inflammatory mediators. However, the gradual decline in these markers over three months indicates that the periodontium can adapt to DME, provided the biological width is respected and smooth margins are achieved.

Goldberg et al.^24^ assert that inflammatory mediators, particularly IL-1 and TNF, are unequivocally associated with inflammation in dental structures, thereby explaining the heightened cytokine levels observed immediately postoperatively.

This outcome is consistent with the research by Know et al.^25^, which indicated that hydroxyl ions produced by these restorative materials modify the redox equilibrium at the lesion site, ultimately leading to chemical tissue irritation and cellular necrosis. Necrotic cells emit minimal quantities of cytokines and other damage-associated signals that promote the clearance of dead or dying cells, resulting in inflammation at the injury site in the absence of pathogens. The significant presence of inflammatory mediators in the GCF of restored teeth, despite the absence of inflammation indicators, may be associated with the reparative process.

Research on cytokines associated with dental caries is limited, particularly regarding gingival crevicular fluid (GCF) from affected teeth. Pulp inflammation resulting from carious lesions is characterized by increased synthesis of proinflammatory cytokines, including TNF-α, IFN-γ, IL-1β, and IL-6. This phenomenon is explained by Celik and Ilday^26^, who observed significant variation in the GCF profiles of IL-6, IL-1, and TNF-α following dental therapy, which they attributed to the disparate local responses elicited by various dental restorative materials.

In the current study, among the tested materials, Tetric N-Flow exhibited the most favorable profile, maintaining the lowest TNF concentrations throughout the trial. In contrast, Beautifil Flow Plus was associated with the highest absolute levels of inflammation. While the exact mechanism underlying this difference remains to be fully elucidated, previous research suggests that fluoride-releasing materials such as Beautifil Flow Plus may influence the local microenvironment differently from conventional composites, as explained by Naoum et al.^27^.

The elevated cytokine levels observed with fluoride-releasing materials in this study are considered preliminary. It is hypothesized that the chemical release from these materials might trigger a localized irritant response as described by Kubota et al.^28^.

A study by Kanjevac et al.^29^ showed that fluoride ions released from materials induce responses in both pulpal and periodontal cells, triggering the release of inflammatory cytokines. Although cytokine levels increased rapidly, they decreased after 1 month but remained higher than in the other groups. The most likely explanation is that the material continues to release fluoride for at least 1 month.

In the current investigation, a noticeable reduction in BOP was observed from the immediate postoperative period (100%) to the 3-month follow-up (60%) across all groups; however, no statistically significant differences were detected among the three materials at either interval. While this uniform downward trend indicates progressive clinical improvement, the persistence of bleeding in the majority of sites (60%) at 3 months warrants cautious interpretation. Consequently, describing the short-term periodontal response as definitive or entirely ‘favorable’ may be premature, suggesting instead that the tissue is still undergoing active remodeling and adaptation.

The positive BOP recorded during the preoperative and immediate postoperative phases can be attributed to preoperative cavitation and food impaction, which trigger localized tissue inflammation. Maintaining excellent dental hygiene was essential to achieving these preliminary reductions. This downward trend in BOP levels aligns with the findings of Oppermann et al.^30^ and Farouk et al.^31^.

Furthermore, the lack of significant differences among the studied materials at 3 months is consistent with Muscholl et al.^22^, who observed no difference in BOP between experimental and control teeth. Mechanistically, bleeding on probing indicates active inflammation at the base of the gingival sulcus, particularly at the marginal edge of the Deep Margin Elevation (DME). In this context, the use of interdental brushes significantly affects the gingival bleeding index, underscoring that regular interdental hygiene, especially in the DME region, is essential for maintaining an inflammation-free condition.

In contrast, research by Ferrari et al.^32^ demonstrated that in most cases of deep-margin elevation, bleeding on probing (BoP) was visible. Positive BoP sites may show various clinical signs. One example occurs when patients strive to maintain hygiene at the deep margins. Additional factors include margin overhangs or underhangs, irregular cervical resin margins, and inadequate control of adhesive and resin-composite flow between the interproximal margin and the metal matrix, all of which are not visible.

The findings of the current study indicated a notable difference in pocket depth between the immediate postoperative period and three months later, with a reduction observed after three months.

Preoperative values exceeded those recorded immediately after and at subsequent intervals, attributable to the impact of cavity preparation, which deepens the cervical cavity margin during the excision of carious lesions. This suggests that polished, smooth, and non-irritating subgingival margins can avert detrimental effects on periodontal tissues. This aligns with the research of Frese et al.^33^ and Farouk et al.^31^.

This is consistent with Peculiar^34^, who explained that a stable periodontal response supports the premise that respecting the biological width during margin elevation is critical for long-term tissue health. By elevating the deep subgingival margin to a more coronal position, the clinician minimizes restorative intrusion into the sulcus, allowing the junctional epithelium and connective tissue attachment to remain intact. Also, Singh^35^ reports that this characteristic of DME aligns with the biological principle of maintaining soft-tissue integrity, which is essential for avoiding chronic inflammation or apical migration of the epithelial attachment.

Conversely, Jepsen et al.^2^ reported that deep subgingival restorations are associated with inflammation and periodontal tissue loss. They noted a lack of data on whether the effects on the periodontium result from biofilm, trauma, dental material toxicity, or a combination of these factors. Additional research corroborates this assertion, particularly regarding the toxicity of subgingivally applied adhesives and composite resins used in this study, Eichelsbacher et al.^36^.

## Conclusion

Within the limitations of this three-month study, all materials were clinically acceptable, though Tetric N-Flow and RM-GI showed a more favorable short-term inflammatory profile than Beautifil Flow Plus. However, due to the limited sample size and short follow-up, no definitive claims regarding long-term periodontal compatibility or clinical superiority can be made.

### Limitation

1. A primary limitation of this study is its short follow-up duration of 3 months. Consequently, the long-term periodontal responses and structural durability of the restorations beyond this period remain unknown, necessitating future long-term clinical trials.
2. Future studies should include a comparison of plaque index scores between the test and control groups.
3. Future studies should include comparisons with other DME techniques, such as thermocut, to elevate the gingival marginal wall and show their effects on periodontal health.

## Supplementary Information

Below is the link to the electronic supplementary material.

Supplementary Material 1

Supplementary Material 2

## Data Availability

The datasets utilized and/or analyzed in the present study are obtainable from the corresponding author upon reasonable request.
