# Supplementary material for: Effect of deep margin elevation with different base materials on periodontal health in a clinical study
Source: Sci Rep. 2026 Jul 16;16:22289. doi: 10.1038/s41598-026-61409-6 (PMC13376392; doi:10.1038/s41598-026-61409-6)
Supplement: Supplementary file 2 — Supplementary Material 2 [file 41598_2026_61409_MOESM2_ESM.docx]

**Effect of Deep Margin Elevation with Different Base Materials on Periodontal Health (Clinical study)**

***Proposal***

Submitted to the Faculty of Dentistry, Minia University, in partial fulfillment of the requirements of a Doctor's degree in Operative Dentistry.

**By**

**Rofida Ragab Mohamed**

B.D.S. (2012)

M.D.S. (2021)

Assistant Lecturer in operative dentistry Department

Minia University

2023

**Supervisors**

**Prof. Mona Ismail Riad**

**Professor of Conservative Dentistry**

**Faculty of Dentistry, Cairo University**

**Dr. Ahmed Abdallah Khalil**

**Prof of oral medicine, oral diagnosis, and periodontology department,** **Faculty of Dentistry, Minia University**

**Dr. Rasha Saad Zaghlool**

**Lecturer of Operative Dentistry,** **Faculty of Dentistry, Minia University**

Date 2-1-2024

**Administrative information:**

1. **Trial Title:**

**Effect of Deep Margin Elevation with Different Base Materials on Periodontal Health (Clinical study)**

**2. Funding:**

The proposed in vivo studies are self-funded.

1. **Roles and responsibilities:**

**3.1. Protocol contributors:**

**3.1.1** **Principal investigator:**

Rofida Ragab Mohamed

- Performs invivo study.
- Ensures specimens preparation throughout the study.
- Collects the baseline and the outcome data of all specimens.

**3.1.2 Chief Supervisor & Scientific Advisor:**

**Prof. Mona Ismail Riad**

**Professor of Conservative Dentistry, Faculty of Dentistry, Cairo University**

- Monitors the whole process from the beginning of the trial and data collection.
- Accesses the final results and data collected from the study
- **3.1.3 Co-Supervisor:**

**Dr. Ahmed Abdallah Khalil**

**Prof of oral medicine, oral diagnosis, and periodontology department, Faculty of Dentistry, Minia University**

**Dr. Rasha Saad Zaghlool**

**Lecturer of Operative Dentistry, Faculty of Dentistry, Minia University**

- Generates a random sequence to enrol the specimens and undergoes proper allocation concealment, implementation, and access to the final data.
- Monitors the process of data collection and checks the ethical standards of the Research Ethics Committee.

**4. Sponsor:**

Minia University.

**Background**

The long-term success of interproximal restorations depends on periodontal health, which is maintained by dental restorations. [1]. For dentists, treating large carious lesions that jeopardize subgingival margins and interproximal contacts is a major issue, as they interfere with multiple stages necessary to achieve the best possible restorations. Effective caries excision, cavity preparation, impression making, rubber dam isolation, gingival bleeding control, appropriate restorative application, removal of excess cement, and biological width compliance are all hampered by deep cervical lesions. The lifespan of the restoration is reduced when saliva, blood, or gingival crevicular fluid contaminates the tooth surface and the restoration margin contact[2],[3].

The main determinant of periodontal health is biological width. Significant cervical defects can result in periodontal deterioration, inflammation, gingival recession, osseous loss, and bleeding, and their treatment frequently depends on the biological width. [4].

The measurement of soft tissue associated with the tooth segment located above the alveolar bone crest is known as biological width. One of the most important surgical procedures for maintaining biological breadth is crown lengthening. Gingivectomy is one of the supplemental operations that is believed to increase procedural efficiency, reposition the margin, and produce a favorable environment for the periodontium. Longer treatment times, higher expenses, discomfort for patients, decreased dental aesthetics, attachment loss, and closeness to root concavities and furcation areas are some of the disadvantages of surgical crown lengthening. [5],[6].

Relocating subgingival margins to supragingival margins and using materials to increase the marginal strength and durability of restorations is known as deep margin elevation, or DME. In some cases, minimally invasive dental management approaches may replace invasive operations, such as crown lengthening, as clinical dentistry places a higher priority on conservation than invasiveness.[7].

Biological factors may cause gingival irritation and compromise the biological width. Operational challenges arise from alterations in tooth morphology, such as deep margins, which may result in enamel loss and impede adhesion to dentin and cementum.[8].

The placement of subgingival margins is associated with detrimental inflammatory periodontal responses resulting from insufficient tooth-restoration interfaces, excessive restoration contouring, challenges in oral hygiene maintenance, heightened pathogenicity of subgingival dental plaque, and a breach of the biologic width principle. Clinical and histological investigations indicate that subgingival restorative margins may induce detrimental tissue effects, despite sufficient bacterial plaque management. Localized gingival inflammation, elevated plaque and gingival index scores, and increased probing depths have been observed around prostheses with subgingival margins in contrast to those with supragingival margins in natural dentition.[9].

The direct composite restoration of teeth is a straightforward, economical, and dependable solution for various clinical scenarios. Progress in adhesive technology, the introduction of modern materials, heightened aesthetic expectations, and the necessity for minimally invasive techniques have resulted in its extensive application in modern dentistry. The application of adhesive materials is complicated in the subgingival region, where overhanging margins are crucial for preserving periodontal tissues. The placement of the cervical margin of restorations is critical, as it may adversely affect biofilm accumulation, irritate gingival tissues, and potentially infringe upon the biological width [10]

The open-sandwich method, which aims to solve sealing issues in deep Class II direct composite restorations, is seen as a forerunner to DME. It fills the cervical area of the cavity with glass ionomer or resin-modified glass ionomer (RM-GIC), leaving some of the material exposed to the oral environment.[11].

The effects of proximal box elevation on the periodontal tissues of premolar teeth filled with direct resin composite were investigated in this study using three base materials: an injectable hybrid composite, a flowable composite, and a resin-modified glass ionomer. The null hypothesis is that there is no discernible difference between deep margin elevation using an injectable hybrid composite and that of RM-GI and flowable composite base materials on the periodontal tissue of direct resin composite inlays.

**Aim of the Study**

This study will be conducted to investigate

The effect of deep margin elevation with different base materials (resin-modified glass ionomer, flowable composite, injectable hybrid composite) on periodontal health (in vivo study).

**Material and Methods**

**Materials:**

- - Resin-modified glass ionomer.
  - Flowable composite.
  - Injectable hybrid composite.

-Nano ceramic composite restoration

**Methods:**

**Sample size calculation:**

Three sets of twelve samples each made up the study's total sample size of N=36. The power surpassed 80%, and the significance threshold was set at 0.05. With a value of 0.5166, the sample size was sufficient, and the confidence interval was 95%. G*Power version 3.1.9 was used to determine the sample size.[12].

**Study setting**

The study will be conducted in the Conservative Dentistry Department, Faculty of Dentistry, Minia University. The researcher will bear ultimate responsibility for all activities associated with the conduct of a research project, including the recruitment of patients, explaining and performing the procedures to them**.**

**Study Design**

A randomized clinical study where participants are randomly assigned to three groups (e.g., one recently introduced injectable resin composite and a standard treatment with RM-GI and flowable composite to compare the effectiveness fairly.

**Ethical regulation:**

This experimental protocol and all investigations comprising human subjects are approved by the Research **Ethics Committee, Faculty of Dentistry, Minia University**.

**Patient selection**

The clinical trial's participants were drawn from the Conservative Clinic at Minia University's Faculty of Dentistry. The lead investigator obtained informed consent for the procedures and explained every aspect of the experiment to eligible volunteers. All participants will be provided written informed consent before enrollment after being fully told about the study's background, including its goals, specific protocols (such as the number of visits), possible advantages, and potential drawbacks

**Inclusion criteria**

Males and females 18 years of age or older who demonstrated adequate oral hygiene, periodontal probing depths of ≤4 mm, full-mouth plaque scores (FMPS), bleeding scores (FMBS) of ≤20%, and at least 2 mm of keratinized tissue were eligible to participate[13].

**Exclusion criteria**

Participants with disabilities, systemic diseases, or serious medical conditions, such as heart disease, diabetes, hypertension, epilepsy, or hematological disorders, as well as those allergic to any research ingredient, were excluded. Patients with severe or active periodontal disease, those who had received therapeutic radiation to the craniofacial region, those who were unable to attend follow-up appointments, and those who had taken part in a clinical trial within three months of the trial's start were all eliminated [14].

**Randomization, allocation of participants, and concealment**

To reduce selection bias, simple randomization was carried out by creating numbers between 1 and 36 using Randomness and Integrity Services Ltd.'s Random Sequence Generator (https://www.random.org/), as demonstrated by Sil et al. in 2019[15].

**Blinding**
Both the patients and the operator are rendered blind. To lessen performance and detection bias, the treatment allocation of each patient was concealed from the three expert evaluators. Additionally, the statistician assigned to analyze the findings will be blindfolded.

**Patients Grouping**

Patients will be divided into three equal groups **(N=12)** according to the type of base material **(B)** used: **(Resin-modified glass ionomer) (B1), (Flowable composite) (B2),** and **(****Injectable hybrid composite) (B3).**

**Table 1: Variables of the Study:**

| **Base materials** | **At baseline** | **After3months follow up** |
| --- | --- | --- |
| **Injectable hybrid composite** | **12** | **12** |
| **Flowable composite** | **12** | **12** |
| **Resin-modified glass ionomer** | **12** | **12** |
|  | **36** | **36** |

**Restorative procedures**

One operator will place all restorations under rubber-dam isolation. A local anesthetic was administered to each patient. Class II mesio-occlusal or disto-occlusal cavity preparations for inlays will be performed. The gingival floor will be located 2 mm below the cementoenamel junction. Following preparation, the cavities were irrigated with water and dried. Flowable composite, injectable hybrid composite, and RM-GI will be applied to each tooth, with each patient receiving only one type of base material.

**Assessment of Gingival and Periodontal Health**

Periodontal health will be assessed by measuring probing depth at the proximal regions of the restorations using a periodontal probe (PCPUNC 15, Hu-Friedy; Chicago, IL, USA). Bleeding on probing (BOP) at designated sites will be recorded in a binary format (“BOP: yes”; “BOP: no”). The gingival bleeding index will be utilized to assess gingival inflammation. Bleeding will be recorded in a binary format (“gingival bleeding: yes”; “gingival bleeding: no”) at the specified locations of the test and control teeth.[20].

**Collection of samples for the measurement of biological markers:**

Samples for biological marker measurement will be obtained from the proximal surfaces of each tooth adjacent to the restored region. Gingival crevicular fluid (GCF) samples will be collected using standard-sized paper strips (Periopaper, OraFlow Inc., NY, USA) after isolating the tooth peripheries with rolled cotton pads to prevent saliva contamination. The concentration of IL-1 and TNF will be measured after restorative procedures and at a 3-month follow-up. Samples will be stored at -20°C for 24 hours at +4°C until biochemical analysis.

**Follow up**

Restorations will be evaluated at baseline (T_0_), 3months (T_1_ ) for pocket depth, bleeding on probing, and concentration of Il-1and TNF at baseline and after 3months follow up.

**Recruitment & Recruitment Strategy:**

Screening of patients who come to the conservative dentistry department seeking dental care will continue until the target population is achieved. The patients will undergo a comprehensive examination and diagnosis using dental charts. Once the patients who are potentially eligible for this study are identified, they will be contacted by the research investigator, who will explain the study and ascertain the patient’s interest. If interested, more detailed evaluations and preparations are made.

**Statistical analysis**

IBM SPSS Statistics (version 26) will be used to conduct statistical analysis. Nominal variables are displayed as frequency and percentage, whereas numerical variables are displayed as mean, standard deviation, and range. P-values below 0.05 (*) were considered significant, and P-values below 0.001 (**) will be considered extremely significant. The following tests are used in this analysis: For parametric variables, the one-way ANOVA is used to compare groups. The difference between measurements made right away and those recorded three months later within each group is evaluated using the Paired t-test. In categorical data, the chi-square test assesses differences across groups.
